# The importance of protein variety in a higher quality and lower environmental impact dietary pattern

**DOI:** 10.1017/S1368980022002221

**Published:** 2022-12

**Authors:** Bradley G Ridoutt, Danielle Baird, Gilly A Hendrie

**Affiliations:** 1 Commonwealth Scientific and Industrial Research Organisation (CSIRO) Agriculture and Food, Clayton South, VIC 3168, Australia; 2 Department of Agricultural Economics, University of the Free State, Bloemfontein, South Africa; 3 CSIRO Health and Biosecurity, Adelaide, South Australia, Australia

**Keywords:** Alternative protein, Australian health survey, Dietary diversity, Dietary guidelines, Life cycle assessment, Meat and alternatives, Sustainable diet

## Abstract

**Objective:**

Eating a variety of nutritious foods is fundamental to good nutrition. However, this principle is challenged when recommendations seeking to improve the environmental sustainability of diets call for avoidance of foods considered to have a higher environmental footprint, such as animal-sourced foods. Our objective was to assess the implications for nutritional adequacy of protein choice across Australian adult diets preselected as having higher quality and lower environmental impact scores.

**Design:**

Each individual diet was assessed for variety of food choice within the ‘Fresh meat and alternatives’ food group defined in the Australian Dietary Guidelines, which includes protein-rich foods such as eggs, nuts, tofu and legumes in addition to animal meats. Diets were grouped according to variety score and whether they included only animal meats, only alternatives or a variety of meat and alternatives. Nutrient content was assessed relative to estimated average requirements (EAR).

**Setting:**

Australia.

**Participants:**

1700 adults participating in the Australian Health Survey

**Results:**

For diets with higher diet quality and lower environmental impact, the likelihood of achieving nutrient EAR significantly increased as variety of food choice in the ‘Fresh meat and alternatives’ food group increased (*P* < 0·001). Variety score and number of serves were also correlated (*r* = 0·52, *P* < 0·001) which is relevant since most diets did not meet the recommended minimum number of serves for this food group.

**Conclusions:**

Greater variety within the ‘Fresh meat and alternatives’ food group is beneficial to meeting EAR and lower environmental impact diets can include three or more selections including foods of animal origin.

There is now an increasing expectation that public health nutrition supports the adoption of sustainable dietary patterns in addition to longstanding health and well-being objectives^(1–4)^. Sustainability is a broad and multi-dimensional concept^(5)^. Already in Australia, and elsewhere, the formulation of dietary guidelines encompasses the availability, affordability, safety and cultural acceptability dimensions of sustainability^(6)^. The additional consideration of environmental sustainability is new and also challenging due to the potential trade-offs with nutrition. For example, many diets with lower greenhouse gas emissions have poorer nutritional and health indicators^(7–9)^. Much of the responsibility for improving the environmental sustainability of the food system rests with food producers. Nevertheless, there is now ample evidence that some dietary patterns have lower environmental impacts than others^(10–13)^, indicating scope for dietary interventions also.

Several approaches are currently being recommended to reduce the environmental impacts of food consumption. The first relates to addressing the high levels of food waste occurring in households and commercial food service^(14,15)^. Though difficult to quantify, this waste could be as high as one-third of purchased food in developed countries^(16)^. The second approach focusses on moderating the intake of energy-dense/nutrient-poor discretionary foods that inflate dietary environmental impacts^(17–19)^. These foods also contribute to excessive dietary energy intake and can displace the adequate intake of healthy core foods, including vegetables^(20,21)^. A third approach involves excluding or limiting animal-sourced foods, particularly those from ruminant livestock such as beef, lamb and goat meats and dairy foods^(22–24)^. This third approach is perhaps the most controversial as it has the potential to impact the nutritional adequacy of the total diet, especially in regions, like Australia, where animal-sourced foods have traditionally formed part of the diet and are important sources of nutrients such as Ca, Mg and Zn that tend to be widely under-consumed^(25–28)^.

In Australia, a subgroup of 1700 adult daily diets was isolated from the National Nutrition and Physical Activity Survey (NNPAS) component of the Australian Health Survey^(29)^. These daily diets were characterised as having higher compliance with Australian Dietary Guidelines^(30)^ and lower environmental impacts. This subgroup of diets is considered important to study because it represents the food choices of Australian adults with more desirable dietary characteristics. As these diets are prevalent in the community, they can be considered culturally relevant and realistically able to be adopted by Australians whose diets are of lower quality and/or have higher environmental impacts. In this study, we further evaluate these 1700 diets by assessing the variety of food choices within the ‘Fresh meat and alternatives’ food group described in the Australian Dietary Guidelines^(30)^. This food group, which includes protein-rich foods such as eggs, nuts, tofu and legumes in addition to meats, is of particular interest because it is most likely to be impacted by sustainability strategies that encourage a transition to plant-based diets. Our goal was to evaluate implications for nutritional adequacy, within the context of the total diet, that could be relevant to inform future dietary guidelines.

## Methods

### Background data

Dietary intake data, covering 9341 Australian adults (19 years and above), were obtained from the NNPAS component of the Australian Health Survey^(31)^ as described previously^(32)^. This survey, undertaken by the Australian Bureau of Statistics, used a 24-h recall process administered though face-to-face interviews by trained assessors, and a complex sampling design to estimate dietary intake for the total population as well as demographic subgroups^(33)^. As part of the national survey, a second 24-h recall was also completed, but this included only 64 % of the original sample and reported significantly lower energy intakes. As this study describes population dietary estimates rather than usual intakes, only data from the first larger 24-h recall were used. In previous studies, each of these diets was scored for level of compliance with the Australian Dietary Guidelines^(30)^ and environmental impact, and cluster analysis was used to isolate a subsample of 1700 higher diet quality and lower environmental impact (HQLI) diets^(29)^. Briefly, diet quality was assessed using the Diet Quality Index of Golley and Hendrie^(34)^. Environmental impact was assessed using currently available life cycle assessment results for food items in the Australian food system^(32,35–37)^. Detailed information about the data, equations and modelling assumptions is available in the associated references. Compared to the population estimate, the HQLI subgroup of adult diets had 39 % higher diet quality score, 53 % lower climate footprint, 24 % lower water-scarcity footprint, 29 % lower cropland-scarcity footprint and 34 % lower pesticide toxicity footprint.

### Analysis of protein variety

For each of the 1700 HQLI diets, the number of servings of the ten different types of foods within the ‘Fresh meat and alternatives’ food group was assessed. This food group includes fish and other seafood; beef and lamb; poultry; pork; eggs; nuts and seeds; tofu and processed meat analogues; legumes; wild meats and offal. This food group excludes processed meats which are considered discretionary foods according to dietary guidelines^(30)^. Intake was assessed in serves, based on Australian Dietary Guideline descriptions^(30)^ because serving size is not defined uniformly across this food group. For example, a serve of red meat is described as 65 g (cooked) and a serve of poultry 80 g (cooked). This compares to 30 g for nuts and seeds, 120 g for eggs and 170 g for tofu^(30)^. A variety score (out of 10) was then calculated for each diet based on the number of different categories included regardless of the amount consumed. For example, if a diet included multiple servings of only chicken, the variety score was 1. If a diet included nuts, eggs and chicken, the variety score was 3.

The 1700 HQLI daily diets were subsequently divided into subgroups based on variety within the ‘Fresh meat and alternatives’ food group (Table 1). In addition, two additional categorisations were performed to assess the nutritional impact of intakes with different combinations of animal and/or plant-Ssourced proteins. The first involved dividing the HQLI daily diets into subgroups according to whether food choices within the ‘Fresh meat and alternatives’ food group were only animal meats, a combination of animal meats and alternatives, or only alternatives (Table 1). Secondly, HQLI daily diets that included a combination of animal meat and alternatives were further divided into subgroups depending on whether they included ruminant meat (i.e. beef or lamb), non-ruminant meat (e.g. poultry, pork, fish), or a combination of ruminant and non-ruminant meats.

**Table 1:** The higher diet quality/lower environmental impact (HQLI) subgroup of adult (19 years and above) daily diets in Australia: intake from the ‘fresh meat and alternatives’ food group (serves) and variety score (*n* 1700)

Subgroup	*n*	Intake of fresh meat and alternatives serves day^-1^	Variety score*
Non-consumer of meats and alternatives (NC)	217	0	0
Variety score of 1 (V1)	845	1·96	1·0
Variety score of 2 (V2)	513	3·16	2·0
Variety score of 3 and above (V3)	125	4·06	3·4
Animal meat only (AO)	756	2·53	1·2
Excluding animal meat (NA)	277	1·50	1·2
Combination of animal meat and alternatives (C)	450	3·30	2·4
Including ruminant meat only (IR)	93	3·10	2·1
Including non-ruminant meat only (ER)	306	3·10	2·2
Combination of ruminant and non-ruminant meat (CM)	41	4·50	3·6

* A variety score was applied to each daily diet^(1–10,51)^ as the number of different types of food from the ‘Fresh meat and alternatives’ food group regardless of the amount consumed. The ten types of food were fish and other seafood; beef and lamb; poultry; pork; eggs; nuts and seeds; tofu and processed meat analogues; legumes; wild meats and offal.

### Nutrient profiling

The nutrient content of each HQLI diet was assessed relative to the estimated average requirements (EAR) published by the National Health and Medical Research Council in Australia^(38)^, using data obtained from the Australian Food Composition Database^(39)^. Statistical analyses were performed using IBM SPSS statistical software package version 26. Population weighting factors were applied to the data prior to running summary estimates, with an additional weighting factor applied to correct for uneven representation of data across days of the week. One-sample *t*-tests were used to test for differences in nutrient composition between subgroups. Correlation analysis was used to assess the relationship between variety score and total intake of ‘Fresh meat and alternatives’ after controlling for total energy intake.

## Results

**Fig. 1:**
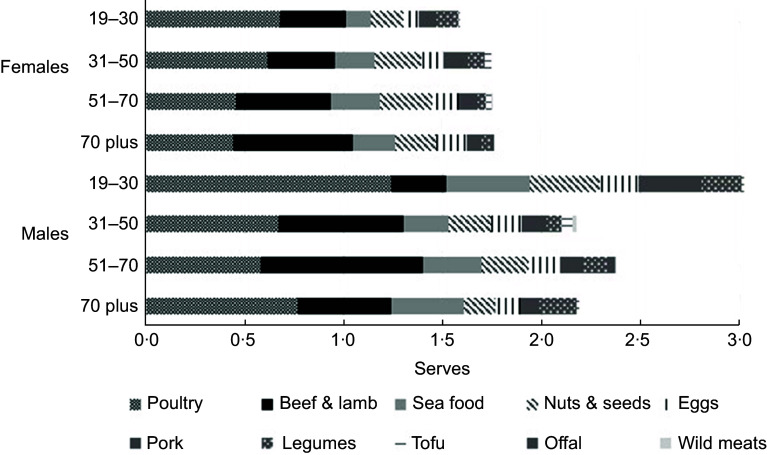
The higher diet quality/lower environmental impact (HQLI) subgroup of adult (19 years and above) daily diets in Australia: Intake from the ‘Fresh meat and alternatives’ food group (average serves) by age and gender (*n* 1700)

For Australian adult diets of HQLI diets, poultry was the most popular protein choice within the ‘Fresh meat and alternatives’ food group defined in the Australian Dietary Guidelines (Fig. 1). This was followed by beef and lamb, and then seafood. The most popular meat alternatives were nuts and seeds, followed by eggs (Fig. 1). Consuming one type of food from the ‘Fresh meat and alternatives’ food group per day was most common (Table 1), and based on previous exploration of these data, this is known to occur predominantly during the evening meal. Greater variety is often associated with inclusion of a ‘Fresh meat and alternatives’ food choice during lunch or during an in-between meal snack (e.g. nuts). Overall, there was a moderate to strong correlation between variety of meat and alternatives reported and total number of serves from the ‘Fresh meat and alternatives’ food group (*r* = 0·52, *P* < 0·001). For HQLI diets that included foods from the ‘Fresh meat and alternatives’ food group, the majority (> 80 %) included animal meat of some type, and of these 28 % included ruminant meat (beef or lamb) (Table 1). Most HQLI diets did not meet the recommended minimum number of serves from the ‘Fresh meat and alternatives’ food group (60·5 %), which ranges from two to three serves depending on age and gender^(30)^.

HQLI diets that did not include foods from the ‘Fresh meat and alternatives’ food group (i.e. non-consumers) had the poorest nutrient composition, with an EAR score of 11, meaning on average this group met 11 of the 16 EAR (Table 2). With increasing variety, significant improvements in nutritional composition were observed (*P* < 0·001), with HQLI diets including three or more types of foods from this food group having an EAR score of 13·2 (Table 2). The nutritional benefits of increased variety were also observed when comparing HQLI diets with and without animal meat, whereby diets without animal meat scored 11·8 compared to diets containing a variety of animal meat and alternatives that scored 13·0. Diets including non-ruminant meats, ruminant meats, and those containing a combination of ruminant and non-ruminant meats scored 12·8, 13·2 and 13·7, respectively (Table 2). Most individual nutrients that differed between groups followed this overall pattern where a diet including a combination of animal meat choices with alternatives scored higher than other variations (Table 2).

**Table 2:** The higher diet quality/lower environmental impact (HQLI) subgroup of adult (19 years and above) daily diets in Australia: percent meeting nutrient estimated average requirements (EAR) according to food choice within the ‘fresh meat and alternatives’ food group (*n* 1700). NC = non consumers; V1, V2, V3+ refer to variety scores of 1, 2 and 3 or above; NA = no animal meat; AO = animal meat only (including fish); C = combination of animal meat and alternatives, ER = excluding ruminant meat; IR = including ruminant meat; CM = combination of ruminant and non-ruminant meats

Nutrient	Percent meeting EAR*									
	NC	V1	V2	V3+	NA	AO	C	ER	IR	CM
Protein	78·5	93·7	97·5	100·0	86·9	97·5	98·6	98·0	100·0	100·0
Thiamine (B_1_)	73·4	74·7	73·5	73·6	71·6	70·5	76·2	72·9	82·3	85·1
Riboflavin (B_2_)	76·2	82·0	87·1	86·4	83·9	79·0	89·4	88·9	89·9	91·0
Niacin (B_2_)†	98·8	99·8	100·0	100·0	99·5	100·0	100·0	100·0	100·0	100·0
Vitamin B_6_	41·7	53·0	59·3	69·6	40·7	60·9	65·3	65·8	49·8	85·1
Vitamin B_12_	64·2	79·4	85·0	92·0	69·2	83·5	90·3	88·4	91·2	99·2
Folate‡	86·0	88·5	86·7	87·2	88·6	85·0	88·8	87·1	94·6	89·2
Vitamin A§	56·0	55·7	56·9	60·0	61·1	57·3	59·4	58·3	62·9	60·5
Vitamin C	82·4	84·7	85·6	81·6	80·9	87·7	84·3	83·3	85·4	87·7
Ca	45·9	34·2	32·7	30·4	45·2	33·0	34·4	34·5	37·3	29·5
Phosphorus	93·1	98·5	99·6	100·0	98·1	99·1	100·0	100·0	99·8	100·0
Zn	33·6	55·5	67·1	68·8	54·3	54·7	67·3	57·4	89·6	88·3
Fe	74·9	81·8	87·9	92·8	85·5	80·9	89·4	87·4	95·4	91·8
Mg	58·0	61·7	71·0	73·6	73·8	57·1	74·6	74·2	75·8	74·7
Iodine	81·2	81·4	83·2	84·8	83·0	78·8	85·1	84·1	85·6	89·4
Se	55·5	79·6	89·7	96·8	59·7	86·9	94·5	96·9	85·1	95·4
EAR score‖	11·0	12·0	12·7	13·2	11·8	12·1	13·0	12·8	13·2	13·7

* EAR defined by the national health and medical research council in Australia^(38)^.

† Niacin equivalents.

‡ Dietary folate equivalents.

§ Retinol equivalents.

‖ Average number of EAR met.

## Discussion

Eating a variety of nutritious foods each day is a universally recognised principle of good nutrition^(40)^ that applies both across food groups and within food groups. This is because each food has its own particular nutritional composition and eating diversely increases the likelihood that all necessary nutrients are obtained. Also, no two individuals are identical in nutrient needs. Variety is so fundamental that it is mentioned almost fifty times in the Australian Dietary Guidelines^(30)^. Yet this is a principle that is being challenged when recommendations to improve the sustainability of the food system call for avoiding or limiting certain foods perceived to be of higher environmental impact, such as replacing animal-sourced protein-rich foods with plant-based alternatives^(41–43)^. Oftentimes, these recommendations are based on evidence comparing the environmental impacts of individual foods deemed to be substitutable. However, frequently this evidence does not consider the implications for overall dietary composition and the impacts of reduced dietary diversity on nutritional adequacy. What this study has shown is that higher quality Australian diets with substantially lower environmental impacts (i.e. HQLI diets) can include higher levels of variety within the ‘Fresh meats and alternatives’ food group defined in the Australian Dietary Guidelines. Furthermore, HQLI diets with higher levels of variety were more nutritionally complete than diets with less variety from the ‘Fresh meat and alternatives’ food group. As such, strategies to achieve healthy sustainable diets in Australia should reinforce, and not undermine, the importance of variety within this food group. HQLI diets with the least likelihood of achieving nutrient EAR were diets that did not include animal meats. It may therefore be necessary for dietary guidelines and regulations concerning food labelling and marketing to more explicitly consider the nutritional risks associated with plant-based substitution of traditional animal-sourced foods, which is an issue that is being increasingly discussed^(44–46)^. Plant-based alternatives may have lower environmental impacts; however, they are not nutritionally equivalent^(47–49)^, and in the Australian context can be lacking in nutrients such as Ca, Mg and Zn that tend to be under-consumed across the population^(50)^.

All that said, it is necessary to acknowledge that this study was based on population estimates of Australian daily diets, not habitual food intake over longer periods. It is likely that for some individuals their food intake and variety may differ from day to day, counteracting to some extent the differences observed in this study. Nevertheless, these results suggest greater variety within the ‘Fresh meats and alternatives’ food group, as recommended by Australian Dietary Guidelines^(30)^, is nutritionally beneficial and that lower environmental impact diets in Australia can include three or more food selections within this food group, including foods that are of animal origin, and those from ruminant livestock production systems. Also, it is important to note that this study has been conducted entirely within the context of the Australian food system, using Australian dietary intake data and Australian nutrient reference values. Therefore, the specific results may not be applicable in other regions, although the general finding that variety in food choices, including protein choices, is beneficial is likely to be generalisable.
